# Experience using of ultrasound guidance pleural tapping in pleural effusion after cardiac surgery in National Cardiac Centre Harapan Kita Hospital. Indonesia (Case Series)

**DOI:** 10.1186/2036-7902-6-S1-A1

**Published:** 2014-01-31

**Authors:** Rita Zahara Ibrahim

**Affiliations:** 1 Post Cardiac Surgery Intensive Care Unit, National Cardiac Centre Harapan Kita, Jakarta, Indonesia; 2 Departemen Cardiology and Vascular Medicine, Faculty Medicine of Fakultas Indonesia

## 

Pleura effusion in post cardiac surgery is the one of problems in management of cardiac surgery patients. Large amounts of pleura effusion may affect the recovery period requiring a longer hospital stay. Early diagnosis and quantification of pleural effusion is important to be ideal postoperative adequate treatment. Ultrasound shows better sensitivity and reliability for diagnosis pleural effusion than physic diagnostic and X-ray. Ultrasound can be repeated serially at bedside without any radiation risk. Procedure perform under ultrasound guided showed a reduction of complication rate.

We report 60 patients with pleural effusion after cardiac surgery. We perform tapping of fluid under guiding ultrasound. Before procedure we calculate amount of pleural effusion and make decision where site of needle will inserted. Regarding the literature we make intraclavicula line as a guided. Tapping procedure will perform if fluid more than 450 cc. Tapping procedure use abbocate needle no 14 or 16fr. Amount of perithoracosistesis fluid is almost same with fluid prediction of before +/- 50 cc, depend on body weight and size of heart if pleural effusion on left thorax. There are not complications after the procedure. Patient experiences were feel convenient and with minimal pain. After procedure patients can complete mobilization and if indication to discharge patients can discharge directly.

## Conclusion

our experience show the ultrasound guided procedure for tapping pleura effusion easy to do, make patient convenient, safe and shorten hospitalization after cardiac surgery.

